# Experiences of Social Harm and Changes in Sexual Practices among Volunteers Who Had Completed a Phase I/II HIV Vaccine Trial Employing HIV-1 DNA Priming and HIV-1 MVA Boosting in Dar es Salaam, Tanzania

**DOI:** 10.1371/journal.pone.0090938

**Published:** 2014-03-06

**Authors:** Edith A. M. Tarimo, Patricia Munseri, Said Aboud, Muhammad Bakari, Fred Mhalu, Eric Sandstrom

**Affiliations:** 1 Department of Nursing Management, Muhimbili University of Health and Allied Sciences, Dar es Salaam, Tanzania; 2 Department of Internal Medicine, Muhimbili University of Health and Allied Sciences, Dar es Salaam, Tanzania; 3 Department of Microbiology and Immunology, Muhimbili University of Health and Allied Sciences, Dar es Salaam, Tanzania; 4 Venhalsan, Sodersjukhuset, Karolinska Institutet, Stockholm, Sweden; The George Washington University Medical Center, United States of America

## Abstract

**Background:**

Volunteers in phase I/II HIV vaccine trials are assumed to be at low risk of acquiring HIV infection and are expected to have normal lives in the community. However, during participation in the trials, volunteers may encounter social harm and changes in their sexual behaviours. The current study aimed to study persistence of social harm and changes in sexual practices over time among phase I/II HIV vaccine immunogenicity (HIVIS03) trial volunteers in Dar es Salaam, Tanzania.

**Methods and Results:**

A descriptive prospective cohort study was conducted among 33 out of 60 volunteers of HIVIS03 trial in Dar es Salaam, Tanzania, who had received three HIV-1 DNA injections boosted with two HIV-1 MVA doses. A structured interview was administered to collect data. Analysis was carried out using SPSS and McNemars’ chi-square (χ2) was used to test the association within-subjects. Participants reported experiencing negative comments from their colleagues about the trial; but such comments were less severe during the second follow up visits (χ2 = 8.72; P<0.001). Most of the comments were associated with discrimination (χ2 = 26.72; P<0.001), stigma (χ2 = 6.06; P<0.05), and mistrust towards the HIV vaccine trial (χ2 = 4.9; P<0.05). Having a regular sexual partner other than spouse or cohabitant declined over the two follow-up periods (χ2 = 4.45; P<0.05).

**Conclusion:**

Participants in the phase I/II HIV vaccine trial were likely to face negative comments from relatives and colleagues after the end of the trial, but those comments decreased over time. In this study, the inherent sexual practice of having extra sexual partners other than spouse declined over time. Therefore, prolonged counselling and support appears important to minimize risky sexual behaviour among volunteers after participation in HIV Vaccine trials.

## Background

Volunteers in phase I/II Human Immunodeficiency Virus (HIV) vaccine trials may be prone to negative socio-behavioural outcomes over time. Although some studies have identified positive social outcomes of participating in HIV vaccine trials such as less risky sexual behaviour among trial participants [1], [2], other studies have documented that during efficacy trials, family and friends perceived that volunteers were HIV-infected or were at risk of HIV infection [3], [4]. In the HIV Vaccine Immunogenicity Study (HIVIS03) trial in Tanzania, volunteers were primed with three HIV-1 DNA injections and thereafter boosted with two doses of HIV-1 recombinant MVA [5]. These volunteers developed HIV vaccine-induced antibodies and cell mediated immune responses after the 5 vaccine doses. Persistence of HIV vaccine-induced antibodies among these volunteers could have long term social consequences when the vaccinees get tested using routine diagnostic HIV antibody assays in other settings and they might end up being labelled as HIV-infected unless HIV polymerase chain reaction (PCR) assay is done to exclude true HIV infection.

Social harm refers to a negative, trial related experience by a study participant, which manifests in psychological, social or physical ways [6]. Apart from being labelled as HIV-infected, individuals may experience other forms of social harm because of their participation in an HIV vaccine trial. Previous studies among volunteers in phase I and II HIV vaccine trials have found a number of experiences of social harm, such as negative reactions from friends, family, co-workers or disturbance in relationships [2], [7], [8]. In Tanzania, significant others (parents, relatives, friends) have been cited as potential barriers towards participation in HIV vaccine trials [9] and this has been the main reason for declining to enrol in HIV vaccine trials [10]. Volunteers in the HIVIS-03 trial experienced diverse negative reactions from friends, family and co-workers. Most of the reactions resulted from the perception that the volunteers were injected with HIV via an experimental vaccine [11]. However, after completing the trial, the volunteers commended the trial team for providing them with regular counselling [11], indicating that follow up studies after completion of vaccine trials would be useful to gain more insight on these social and behavioural issues.

Participation in HIVIS03 trial required volunteers to be at low risk of acquiring HIV infection, and they were expected to be able to adhere to safe sexual practices throughout the trial. The HIVIS03 volunteers were assessed and categorized as being of a low risk group before the trial, but after completing the trial some volunteers confessed that they were actually at high risk before enrolling in the trial [11]. Nevertheless, they appreciated the trial team for encouraging them to change towards less risky sexual behaviour [11]. Taking into account the reported sexual behaviour before enrolling in the trial, volunteers may encounter risk compensation by believing in the protective effect of the candidate vaccine that may encourage increased risky sexual behaviour after the trial. A previous study among HIV-1 seronegative men and women in Canada revealed that a few non-enrolees in a Phase IIb prophylactic HIV vaccine trial articulated their concern about engaging in increased high-risk behaviours if they enrol in the trial [12]. Prolonged follow up of HIVIS-03 volunteers was considered important so as to assess possible changes of sexual behaviour over time. Therefore, the current study aimed to study the persistence of social harm and change in risky sexual practices over time among HIVIS03 volunteers following completion of the trial.

## Methods

### Ethics Statement

Ethical approval was obtained from the Senate Research and Publications Committee of the Muhimbili University of Health and Allied Sciences, Dar es Salaam, Tanzania. Written informed consent to participate in the study was obtained from the individual participants prior to enrolment in the study.

### Study Design

This was a descriptive prospective cohort study conducted among the HIVIS03 volunteers between 2 and 3 years following the second HIV MVA boost. The social behavioural follow up study over the two follow up visits was conducted in October/November 2010 and March 2011.

### Study Setting

The study was conducted in the HIV Vaccine Clinical Trial Unit at Muhimbili National Hospital in Dar es Salaam, Tanzania.

### Study Population

The study population comprised of volunteers enrolled in a double blind Phase I/II HIV vaccine trial (HIVIS03) conducted between 2007 and 2010. Details of the HIVIS-03 trial have been published elsewhere [5]. Only 60 volunteers were enrolled in the HIVIS-03. This small sample size is based on the criteria set forth for a phase I/II HIV vaccine trial in that a sample of 50–100 participants is considered adequate [13]. Of the 60 volunteers, 38 received three doses of HIV-DNA priming followed by two doses of HIV-1 MVA boosting. The rest received placebo and were thus excluded from this follow up study. The first and the last volunteers received the second MVA vaccination on 3^rd^ February 2009 and 8^th^ July 2009, respectively, and the study was closed on 19^th^ February 2010. Thirty three of the 38 volunteers participated in the current study. The rest were not in Dar es Salaam during the data collection period. During the follow-up period, the nurse-counsellors at the trial site continued to provide all volunteers with counselling services. At least five sessions of counselling on importance of adhering to safe sexual practices were provided during the follow-up period.

### Recruitment and Data Collection

Each participant was followed up twice at 17–22 and 30–35 months after the second HIV MVA vaccination. During each visit, clinical and laboratory evaluations were done followed by social behavioural assessment. Findings from the clinical and laboratory evaluations will be reported separately. In social behavioural assessment, a structured questionnaire was administered by nurse counsellors to assess social harm, and risky sexual behaviour. The main survey questions were: (1) Have you come across negative comments about your previous participation in the HIV vaccine trial from any of the following people? This question was followed by a list of types of people as shown in **Table 1**. (2) If yes in any of the above categories, what type of comments among the following options, did they use towards you? Also this question was followed by examples of comments as shown in **Table 2**. (3) Have you been diagnosed with sexually transmitted infections in the last six months? (4) Have you had penile vaginal sex with a partner of unknown HIV status in the last six months? (5) If yes to question 4, did you use a condom during the last time you had sex with such a partner? (6) Do you have a regular sexual partner other than spouse or cohabitant? All questions reflected the volunteers’ perceptions during the trial. During the second follow up visit, participants were asked similar questions. The questions were constructed from the findings of qualitative evaluation of HIVIS03 volunteers [11].

**Table 1:** Negative comments/reactions from various people (Insert tick [√] in the appropriate box(s)).

S/N	Type of a person	Yes	No	Don’t remember
1	Colleagues at workplace			
2	Friends in the community			
3	Blood relatives (brother(s) or sister(s)			
4	Close family member whom you are currently staying with			
5	Father			
6	Mother			
7	Guardian			
8	Boss/supervisor at your workplace			
9	Health care provider(s)			
	Doctor(s)			
	Nurse(s)			
	Laboratory technologist(s)			
	Counsellor(s)			
10	Others (specify)			

**Table 2:** Types of negative comments/reactions from other people (Insert tick [√] in the appropriate box(s)).

S/N	Type of phrase/words	Yes	No
**1**	Sigma e.g. Other people telling you ‘You are infected with HIV because you took part in an HIV vaccine research/trial’		
**2**	Jokes e.g. ‘Phrases intended to make fun of you in front of other people who are not familiar with the HIV vaccine trial’		
**3**	Discrimination e.g. Colleagues not wanting to involve you in certain issues because of your previous participationin an HIV vaccine trial		
**4**	Failure to establish new sexual relationship e.g. A girlfriend/boyfriend stopping relations with you after learningthat you had previously been enrolledin an HIV vaccine trial		
**5**	Termination of sexual relationship e.g. Husband or wife separated from you because you previously took partin an HIV vaccine trial		
**6**	Lack of psychological support e.g. When you are sick or in need of support from the family		
**7**	Mistrust of HIV vaccines from the surrounding community e.g. ‘People telling you that, this vaccine you were involvedin will affect you in future’		
**8**	Other phrases, specify		

### Analysis

SPSS for windows version 20.0 was used. Frequency distribution of socio-demographic characteristics was determined. We used McNemars’ chi-square (χ2) to test the association within-subjects because the same individuals were measured (or surveyed) twice, and thus the same variables were matched. In McNemars’ chi-square, the degree of freedom is 1, and the critical value is 3.84 (from the chi-square table). Therefore in this study if the calculated value exceeded 3.84, then there was a significant difference between first follow up and second follow up responses. Significance of differences between proportions was ascertained through p-values. A value less than 0.05 was considered significant. Regardless of the presence or absence of differences, responses in both the first follow up and the second follow up are presented for discussion purposes. However, we present the P-value of significant results only. All published and unpublished data about HIVIS03 may be accessible on request from the authors.

## Results

### Study Participants

Of the 33 study participants, the majority (82%) were men, married (58%); and educated to four years of secondary education (49%). Two participants changed their marital status from cohabiting to single/separated/widowed over the two follow-up periods. The rest maintained their socio-demographic characteristics over the first and second follow-up periods (Table 3).

**Table 3:** Baseline characteristics of the study participants.

Variable	No (%)
**Sex**	
Male	27 (81.8)
Female	6 (18.2)
**Marital status**	
Single/Separated/Widowed	10 (30.3)
Cohabiting	4 (12.1)
Married	19 (57.6)
**Education**	
Seven years primary	11 (33.3)
Four years secondary	16 (48.5)
Advanced	6 (18.2)
NB: Mean age in years = 34 (24–51)	

### Comments Towards the HIVIS03 Trial Participants

The participants encountered negative comments from various individuals but these were more from amongst colleagues. The comments from the colleagues towards the volunteers changed significantly over the two follow-up periods (p<0.001). The colleagues were more likely to give negative comments to the volunteers during the first follow-up (57.6%) than during the second follow up (27.2%). Siblings, close family members, parents or guardians, seniors at work and health care providers were less involved in giving negative comments to the participants (Table 4).

**Table 4:** Negative comments/reactions from various people about participants’ involvement in an HIV vaccine trial.

	First follow-up		Second follow-up		
Category of a person	Yes (%)	No (%)	Yes (%)	No (%)	McNemars’ χ2 p-value
Colleagues	19 (57.6)	14 (42.4)	7 (21.2)	26 (78.8)	8.72***
Friends	6 (18.2)	27 (81.8)	2 (6.1)	31 (93.9)	0.96
Blood relatives	1 (3.0)	32 (97)	2 (6.1)	31 (93.9)	0.0
Close family member	4 (12.1)	29 (87.9)	1 (3.0)	32 (97)	0.54
Father	1 (3.0)	32 (97)	0 (0.0)	33 (100)	0.06
Mother	1 (3.0)	32 (97)	0 (0.0)	33 (100)	0.06
Guardian	1 (3.0)	32 (97)	0 (0.0)	33 (100)	0.06
Boss	1 (3.0)	32 (97)	1 (3.0)	32 (97)	0.00
Doctors	1 (3.0)	32 (97)	1 (3.0)	32 (97)	0.00
Nurses	1 (3.0)	32 (97)	0 (0.0)	33 (100)	0.06
Laboratory technician	1 (3.0)	32 (97)	0 (0.0)	33 (100)	0.06

Significance marked as: ***P<0.001.

### Types of Negative Comments

Most of the negative comments towards the participants were associated with discrimination, stigma and mistrust towards the vaccine in the HIVIS03 trial. Discrimination from colleagues towards the volunteers changed significantly over the two follow-up periods (p<0.001). The colleagues were more likely to discriminate against the volunteers during the first follow-up (63.3%), while none of the volunteers was discriminated against during the second follow up. Also stigma and mistrust of the vaccine trial towards the volunteers changed significantly over the two follow-up periods (p<0.05) (Table 5).

**Table 5:** Types/nature of negative comments/reactions received from other people (non- vaccine participants) and the effects of words in the relationships.

	First follow-up		Second follow-up		
Nature of comments	Yes (%)	No (%)	Yes (%)	No (%)	McNemars’ χ2 P-value
Discrimination	21 (63.6)	12 (36.4)	0 (0.0)	33 (100)	26.72***
Stigma ‘you are infected with HIV because you took part in the vaccine research’	17 (51.5)	16 (48.5)	7 (21.2)	26 (78.8)	6.06*
Jokes about the vaccine	12 (33.3)	21 (66.7)	5 (15.2)	28 (84.8)	2.96
Mistrust towards the vaccine in the trial	11 (30.3)	22 (69.7)	2 (6.1)	31 (93.9)	4.9*
Failure to establish new sexual relationship	1 (3.0)	32 (97)	0 (0.0)	33 (100)	0.06

Significance marked as: ***P<0.001; *P<0.05.

### Sexual Practices and Outcomes

There was no difference between first and second follow up in aspects of sexual practices, except in terms of having a regular sexual partner other than a wife or husband. Having a regular sexual partner other than the spouse or cohabitant changed significantly over the two follow-up periods (p<0.05). Participants were more likely to have a sexual partner other than the spouse or cohabitant during the first follow-up (30.3%) than during the second follow-up (6.1%) (Table 6).

**Table 6:** Sexual practices and outcomes within the previous six months.

	First follow-up		Second follow-up		
Response	Yes (%)	No (%)	Yes (%)	No (%)	McNemars’ χ2 P-value
Diagnosed with sexually transmitted infections	1 (3.0)	32 (97)	0 (0.0)	33 (100)	0.06
Had penile-vaginal sex with a partner of unknown HIV status	5 (15.2)	28 (84.8)	3 (9.1)	30 (90.9)	0.24
• Used condoms with a partner of unknown HIV status	4 (80.0)	1 (20)	2 (66.7)	1 (33.3)	0.54
Has regular sexual partner other than spouse or cohabitant	10 (30.3)	13 (69.7)	2 (6.1)	19 (93.9)	4.45*
Sexual partner inject drugs	1 (3.0)	32 (97)	0 (0.0)	33 (100)	0.06

Significance marked as: *P<0.05.

### Prediction of Safe Sexual Practices

Consistently, the majority (76%) of the participants stated that they were very sure of the intention to practice safe sex throughout their lives. However, a few (21%) were not sure at all if they could practice safe sex forever.

## Discussion

Prolonged socio-behavioural follow up of participants after completing scheduled visits in an HIV vaccine trial is important, especially in settings where the conduct of such trials is still a rarity. In this study, participants experienced socio-behavioural changes over the follow up periods. The results showed the presence of and a significant decrease of negative reactions such as discrimination and stigma from their colleagues. Also there was mistrust of the vaccine trial from the surrounding community but it also decreased over time. In contrast, there was no significant difference of negative reactions from family members. Also during follow up, a number of sexual partners outside a steady relationship decreased. However, some volunteers were not sure at all to be able to practice safe sex forever. It was encouraging that consistently, the majority of the study participants were very sure to be able to practice safe sex throughout their lives.

The persistence of minimal negative reactions from the family members imply that volunteers were accepted in their comfort zones, the family where they often obtained a range of psychological and social support. Nevertheless, the fact that negative reactions from colleagues decreased during the second follow up implies that co-workers may have continued to understand the meaning and safety of participating in an HIV vaccine trial. This contradicts previous findings among volunteers in the same trial that indicated that significant others including co-workers were a major source of negative reactions towards the volunteers during the conduct of the trial [11]. Others have also found negative reactions of co-workers were the most commonly reported incidents [7]. Discrimination and stigma are significant forms of social harm among volunteers who participate in HIV vaccine trials. In the present study, there was a significant decrease in discrimination and stigma towards the participants indicating that social harm among volunteers may decrease over time. Such decrease might have been due to firstly that over time co-workers become habituated to their colleague’s participation in the trial and gain a clearer understanding of the trial which helps to decrease negative responses, but also over time the proximity of the trial in family and co-workers everyday lives becomes further removed, less relevant and they therefore are less likely as time goes on to refer at all, either positively or negatively to their colleague’s participation.

While mistrust in vaccine trials has been documented for more than a decade, it has gained more attention during the conduct of HIV vaccine trials given the nature of the disease. However, in this study, the decrease in mistrust towards HIV vaccine trial implies that the community may have increasingly understood the logic behind the conduct of HIV vaccine trials. Alternately the volunteers may have demonstrated confidence and assertiveness while dealing with the mistrust as revealed in the previous study [11].

Sexual behaviour among volunteers in HIV vaccine trials has been evaluated in the previous studies [1], [14], [15], [16], [17]. In this study, having a regular sexual partner besides the wife or husband was reported by only 6.1% of the volunteers compared with the findings in the same police cohort in the mid nineties in whom 36.2% (212 of 585) had admitted to have had at least one extramarital sexual intercourse in the previous 3 months at which occasion condoms were not used during the encounters by 178 of 212 (84.0%) individuals [18]. The current result suggests that prolonged follow up counselling is useful towards such a significant decrease in number of volunteers who reported to have had extra sexual partners during the second follow up. A previous study in the same HIVIS03 vaccine trial population showed that a majority (58.2%) of study subjects were having a regular sexual partner, other than wife/husband [19]. Nevertheless, volunteers in the HIVIS 03 trial were assessed and categorized as a low risk group for acquiring HIV infection; this was proven true in the follow up results as all the volunteers who had received all the five doses of candidate vaccine products were confirmed by HIV-1 DNA PCR assay to be HIV-uninfected about two years after the second HIV MVA boost (un published data). Similarly, in another study, volunteers reported relatively low levels of risk behaviours at baseline and at the follow-up visits [20]. Also in another study there was no increase in risky behaviour during a follow up study among intravenous drug users in HIV vaccine trial [17] Therefore, it was not surprising that consistently, the majority of the HIVIS 03 volunteers thought they would be able to practice safe sex throughout their lives. However, caution should be taken when interpreting these results. The low risk-taking could be due to the participants’ selection bias. On the other hand, the low risk-taking behaviour may represent reporting bias, in which study participants falsely report safer sexual practices to please the trial team. Albeit, volunteers were consistently reminded about the importance of adhering to safe sex practices [11], the issue of sexual behaviour is controversial because during this follow up, a few volunteers did not use condoms with a partner of unknown HIV status. This could be due to risk compensation shortly after the trial implying that volunteers might have been thinking they were protected. Although the results from this study do not directly confirm the existence of risk compensation following phase I/II HIV vaccine trials, the study adds contextual insight about sexual behaviour of volunteers shortly after the trial.

### Limitations

This is a small scale study conducted among volunteers who participated in the phase 1/11 HIV vaccine trial. Prolonged follow up study was planned to support the volunteers but also to analyse change of various parameters over time including socio-behavioural aspects. Some issues might have not been revealed in this study because of the nature of study design, population and sample size. The study design: The variables were selected based on the initial qualitative study of which we only focused on the revealed aspects of socio-harm among these volunteers [11]. Therefore, the questions were designed from the volunteers’ perception. Possibly the questions used may have affected the reported results because of social desirability bias. That means for example, there is a risk of sexual practices reported not representing the actual sexual practices. In addition, social desirability bias may obstruct the results because the information we report here is based on reported as opposed to actual behaviours. Although the measurements for sexual behaviours in this study are unlikely to represent the true sexual behaviours of the cohort, we think that the obtained information may be useful to design a future study. However, emphasis should be on using appropriate validated measurements. The study population was prone to selection bias given that the volunteers were defined as a low risk group from the start. Therefore, the study design was not optimal since the sample generates selection bias. While we cannot generalize the results beyond the studied sample, the knowledge gained is important for future HIV vaccine trials in Tanzania.

## Conclusions

Follow up of trial participants after completing the scheduled trial visits is important because some volunteers may continue to suffer from social harm. Participants in phase I/II HIV vaccine trials are likely to face negative reactions after completing the trials, but these reactions may decrease over time. Also what we are observing from these results is that risky sexual behaviours may persist for some volunteers. It follows that; prolonged consistent counselling among the volunteers may identify types of social harm and risky sexual behaviour. Also, this kind of follow-up is important because participants in HIV vaccine trials may face different challenges that the trial team may not be aware of. Therefore, it is high time for those planning future HIV vaccine trials to continue to support the volunteers for a period of time after the completion of the trial in order to address possible socio-behavioural challenges.
